# Behind the thinking of attention disorder

**DOI:** 10.3389/fpsyg.2025.1358405

**Published:** 2025-04-16

**Authors:** Daniela Zamboni, Vicki Snyder

**Affiliations:** ^1^Instituto de Potencialidades Humanas, Campo Grande, Brazil; ^2^The MINDCAP Center, Fort Wayne, IN, United States

**Keywords:** cognition, attention disorder, information processing, cognitive modifiability, neuroplasticity

## Abstract

This article presents an innovative exploration of attention disorders by focusing on their cognitive structure and the power of neuroplasticity, moving beyond traditional models that primarily emphasize lessening behavioral manifestations. By employing Reuven Feuerstein’s theory of Structural Cognitive Modifiability, it examines the cognitive architecture of individuals with attention disorders, emphasizing the dynamic interplay between cognitive processes, neuroplasticity, and behavioral outcomes. The paper identifies distinct cognitive patterns influencing focus, organization, and planning, which manifest in diverse presentations of inattention. It highlights the potential for precise, individualized cognitive interventions to address inefficiencies in information processing, foster self-regulation, and enhance brain adaptability. By addressing the origins rather than the complaints this approach offers a transformative framework for enhancing self-regulation, cognitive adaptability, and overall learning outcomes in individuals with attention disorders.

## 1 Introduction

Characterized by a range of cognitive challenges, attention disorder has garnered significant attention and widespread publicity. The aspects most attributed to this condition are weaknesses in maintaining or sustaining attention over time and across tasks. This disorder, diagnosed as ADHD, has also been the subject of extensive research and debates, emphasizing its impact on individuals. The American Psychiatric Association (2022) defines ADHD as a persistent pattern of inattention, hyperactivity-impulsivity, or a combination that interferes with functioning or development, and symptoms exhibited in two or more settings (e.g., at home, school, or work) with direct negative impacts on areas such as social, academic, or occupational functioning. It is categorized as a neurodevelopmental disorder and generally classified as one of the following:

- Predominantly inattentive presentation
- Predominantly hyperactive presentation
- Mixed presentation, showing characteristics of both

Using data from a 2022 national parent’s survey, The Centers for Disease Control and Prevention (n.d.) (cdc.gov) illustrates the prevalence of ADHD. 11.4% of children, or 7 million, have received a diagnosis of ADHD. This is an increase of 1 million children compared to 2016. According to this same survey, about six in 10 children with ADHD had moderate or severe ADHD, and this was most significant among those who had a co-occurring condition. Severe ADHD was noted with a co-occurring condition which includes learning disorders, anxiety, depression, and behavioral or conduct problems. The CDC reports that ADHD can often be managed with the right treatment and refers to medication, behavior therapy, or a combination, as recommendations (cdc.gov). Other current treatments used to manage its effects are neurofeedback (Monastra, 2022), and lifestyle changes such as nutrition, exercise, and sleep (Nigg, 2024).

A recent report by researchers from the National Institutes of Health (NIH) identifies a link between attention-deficit/hyperactivity disorder (ADHD) symptoms and patterns of interaction between the brain’s frontal cortex and deeper regions involved in information processing. The study, published in the American Journal of Psychiatry, drew from imaging data of more than 8,000 youths, both with and without ADHD. The researchers investigated how brain connectivity relates to ADHD characteristics. Their findings revealed that youths with ADHD exhibited stronger connectivity between subcortical areas associated with learning, movement, reward, and emotion, and frontal regions of the brain that govern attention and the suppression of impulsive behaviors (National Institutes of Health, 2024).

While current literature points overwhelmingly to different wiring for those with an attention disorder, this does not preclude the reality of neuroplasticity. According to Psychology Today, neuroplasticity is the brain’s capacity to continue growing and evolving in response to life experiences. Plasticity is the capacity to be shaped, molded, or altered; neuroplasticity, then, is the ability of the brain to adapt or change over time, by creating and building new synaptic connections (Psychology Today, n.d.).

Neuroplasticity is the brain’s capacity to continue growing throughout life by reorganizing neural networks, rewiring them in a way that differs from how they previously functioned, meaning the brain can change, thus refining its very structure. According to Doidge, 2007, neural activities both tracked and identified have been discovered to show an effect on neurons, glial cells, and vascular cells. The ability to change allows for the development of new cognitive skills as well as the calibration of existing ones, making them more efficient, which in turn changes both thinking and brain functioning. This dynamic ability of the brain to reorganize its cortical structure enables mental and behavioral changes. This has important implications for a wide range of behaviors.

According to Kleim and Jones (2008):

“Over the last several decades, neuroscience research has begun to characterize the adaptive capacity of the central nervous system (plasticity). The existing data strongly suggest that neurons, among other brain cells, possess the remarkable ability to alter their structure and function in response to a variety of internal and external pressures, including behavioral training. We will go as far as to say that neural plasticity is the mechanism by which the brain encodes experience and learns new behaviors” (p. 225).

Reuven Feuerstein, an Israeli psychologist, in his theory of Structural Cognitive Modifiability (Feuerstein et al., 2015a, pp. 87–88). According to Feuerstein, “Today when we speak about modifiability, we are referring to a process that is more important than ever thought before, for modifying not only our behavior but also our operational thinking and extending to the modifiability of the neural system itself.” Feuerstein emphasizes, “the brain not only produces certain behaviors but also is actually shaped by the behaviors that the individual imposes on the brain.” Combined with the concepts of neurogenesis (the process of generating new neurons) and epigenesis (the coaction of genes and environment to shape the individual), it is possible to embrace the fact that the brain is a flexible and plastic organ, making wiring and rewiring possible throughout the lifetime of an individual.

This change may be directed through activities imposed on the individual in appropriate cognitive intervention programs with carefully guided coaching, which is meaningful to the learner, creating awareness. Kleim and Jones (2008) proposed nine effects of elements that promote neural plasticity, later reframed by Falik (2019) with clinical and didactic experience adding three additional effects of elements promotive of neural plasticity. The concept of the “consciousness/awareness effect” is delineated, wherein it is described as an educational mechanism that enhances the learner’s awareness of how cognitive functions influence behavioral outcomes and possible interventions. This effect plays a pivotal role in strengthening other alterations in cognitive functioning, thereby fostering increased engagement and a readiness to embrace frustration and the necessity for heightened effort in response to varying situational demands.

Purposeful structural cognitive modifiability occurs with clear-cut targeting of what needs changed, activating and stimulating specific cortical brain functions with a high-level of repetition and intensity using specific tasks and mediation by the professional. This approach requires time, effort, and precise activities and is powerful for changing behaviors in an individual and affecting the neural substrata itself.

A pilot study conducted by The MINDCAP Center in Fort Wayne, IN, in 2018, in collaboration with Parkview Regional Health Systems used this approach to determine the ability to affect ADHD using The Feuerstein Method. The research group received intensive and integrated services to teach them about their brain’s neuroplasticity and help their shift to a growth mindset, as well as two Feuerstein programs.

The areas addressed for mediation of improvement for the study were focus, impulse control, and planning behavior. The control group received learning style-based tutoring in reading, math, and study skills by university students. Both groups received the same number of hours of services; pre-, mid, and post-test assessments were taken. These assessments consisted of Conner’s Assessment for ADHD; the Behavior Rating Inventory of Executive Functions (BRIEF); and the Disruptive Behavior Stress Inventory (DBSI).

The research group showed improvement to a greater degree than the control group in working memory, and a significant improvement in the impact a child has on a family due to disruptive behavior compared to the control group (Zehr and Bergeron, 2019). *Another way for ADHD: Application of The Feuerstein Program as a Non-Pharmacological Approach to Improving Symptoms of ADHD* [Unpublished Manuscript].

This study provides clear evidence of neuroplasticity in action. By employing targeted cognitive interventions and guided mediation, it demonstrated that purposeful activities could lead to meaningful changes in brain function and behavior. These findings reinforce the concept of the “consciousness/awareness effect,” emphasizing how increased awareness of cognitive processes enables individuals to embrace growth and adaptability. This study confirms that neuroplasticity, when harnessed through precise and intentional methods, is a powerful mechanism for fostering cognitive and behavioral transformation.

The purpose of this article is to propose understanding the roots of patterns in the cognitive structure from the perspective of Feuerstein (1980) that brings inattention, thus allowing for the development of a precise cognitive intervention to impact the “wiring” of the brain.

## 2 Grounding

As cognitive educators and coaches, the authors have worked with clients with a reported diagnosis of attention disorder, either singularly or as a co-diagnosis. The base of work is to understand the cognitive functional qualities and nature of the individual from a cognitive behavioral, functional perspective.

The data used to delineate the findings in this paper is the result of over 100 assessments by author Zamboni using the two tools described below.

One of the tools used in the work was the Learning Propensity Assessment Device- LPAD, a dynamic assessment developed by Feuerstein et al. (2002) to “understand how people process information, and identify the open gate to their comprehension and learning processes” (p. 159). The open gate is accessed by considering the level of efficiency of cognitive functions in the cognitive platform. Feuerstein’s theory supports that learning style is defined as how information penetrates the person’s thinking structures and generates a structural modification. This is reflected in his Theory of Cognitive Structural Modifiability.

The assessment brings information about the dynamic of the cognitive functions in the individual’s cognitive structure. From it, it is possible to understand their level of efficiency, inefficiency, or even deficiency (when the cognitive functions do not appear automatically in their thinking). From this analysis, we can infer the architecture of thinking of the individual and, consequently, how their processing of information is built and then, from it, plan a precise intervention.

Another tool used was Neethling Brain Instruments (NBI), an assessment whose origins came from the studies of Ned Hermann, later continued by Kobus Neethling. Herrmann (1988) proposed the theory of Whole Brain Thinking in which he affirms that according to the way an individual reasons, there are certain areas of the brain that they favor when thinking. His research on the brain has led to an understanding that each of us has a specialized thinking preference that affects the way we take in and process information.

## 3 The cognitive structure

The cognitive structure is responsible for sustaining the modus operandi through which the individual accesses, processes, and responds to the various sources of stimuli received.

The cognitive structure can be viewed as not static but an open, dynamic system that can be developed, shaping the brain. According to Feuerstein, there is a relationship between cognitive modifiability and neuroplasticity. In his book *Changing Minds and Brains* he affirms that the change in the cognitive structures brings not only a change in mental, operational, and emotional behavior, but also affects the brain structure itself, generating new neurons, creating new links in the brain, and producing new cognitive structures for the need of adaptation of the individual (Feuerstein et al., 2015a, p. 88).

When the data enters the cognitive system, it is just data. There is no interpretation at this point because the cognitive structure performs the interpretation. Through that structure, mental operations are performed, supporting the reasoning (Feuerstein et al., 2015b, p. 89). The thinking process demands mental operations, a topic deeply discussed by Jean Piaget, a Swiss psychologist known for his work on the study of cognitive development, and fundamental to understanding cognition and the mental process resulting from it. According to Piaget (1952), as cognitive structures develop, these operations grow in complexity, enabling a progression from concrete thinking to more abstract reasoning.

The cognitive structure is the matrix that promotes mental acts manifested through mental operations. Mental operations are strategies or rules used to organize the various sources of information accessed. Feuerstein researched cognitive development seeking to understand the prerequisites for developing mental operations. This led to the development of the Theory of Structural Cognitive Modifiability (Feuerstein, 1980). He believed that cognitive modifiability created a change in the brain, thus altering it. He argued that the brain was moldable, a fact later confirmed by neuroscience and now known as neuroplasticity.

According to Feuerstein et al. (2006) for mental operations to be established, they demand several cognitive functions: “Cognitive functions are the mental conditions essential to the existence of thinking operations and any other behavior function. The key word in this definition is condition. Cognitive functions are defined as conditions in which mental operations are performed”.

This finding grounded the Theory of Structural Cognitive Modifiability through the delineation of 27 cognitive functions. The effectiveness of mental operations depends directly on these supporting cognitive functions, which then act in combination to provide the necessary conditions for the functioning and modifiability of the cognitive structure.

The various cognitive functions are organized into three different phases presented below (Feuerstein et al., 2006, p. 135):

1. Input phase - The initial phase of mental activity is responsible for searching for and gathering data into the cognitive system.
2. Elaboration phase - The processing phase transforms information into distinctive and organized knowledge. This elaboration turns data into knowledge and creates patterns and relationships among the data in the system. It is the core of the cognitive process, demarcating the core of the individual’s reasoning.
3. Output phase – This phase of the response is the result of the mental construction carried out earlier in the input and elaboration phases.

All phases of the mental act are composed of essential and non-linear specific cognitive functions and can present themselves at various stages of development. They may be highly efficient, register degrees of inefficiency when they seem ineffectively employed in problem-solving, or even present themselves as deficient when they do not appear spontaneously in the individual’s cognitive process (Feuerstein et al., 2002, p. 136).

Table 1 shows 27 developed cognitive functions, grouped according to the three phases of the mental act (input, elaboration and output), to assist in the microtomy of the cognitive structure.

**Table 1:** Developed cognitive functions.

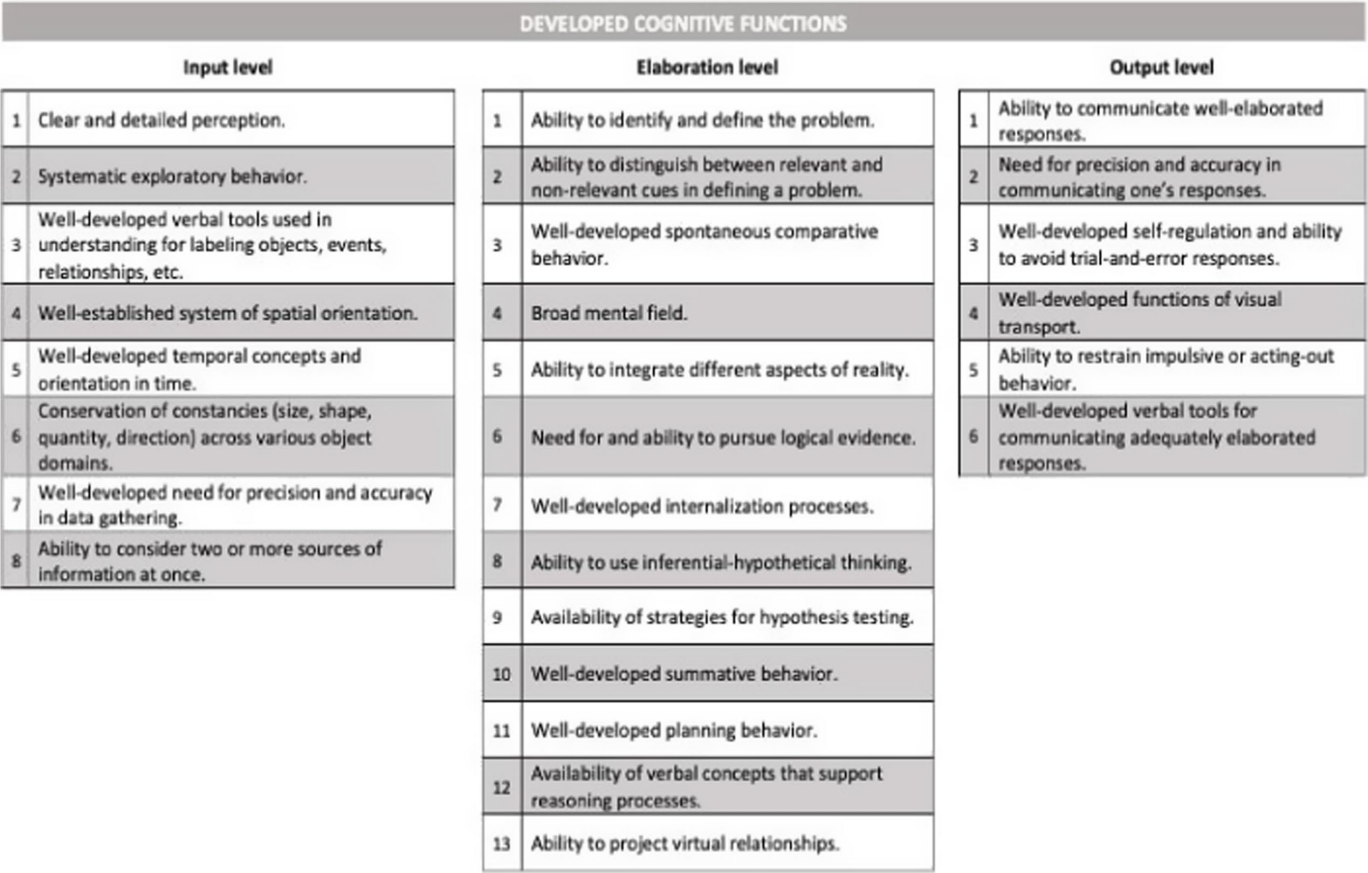

Note: The initial application of the cognitive functions to address modifiability was addressed by Feuerstein and his colleagues through the framing of them as impaired or deficient. Feuerstein (1980)

Source: (Feuerstein et al., 2006).

## 4 The physiology of cognition

The above cognitive functions represent the physiology of cognition. From their level of functioning, it is possible to infer the individual’s processing of information and, consequently, the grounding of their comprehension. Once the dynamic of the cognitive functions is identified and understood it is possible to determine the individual’s architecture of thinking at the time of assessment.

The architecture of thinking is a proposed analogy to explain that every person has a unique thinking framework. Unlike a building, whose structure follows a specified design, our mind’s structure is dynamic and results from the organization of our thinking framework.

**Figure 1:**
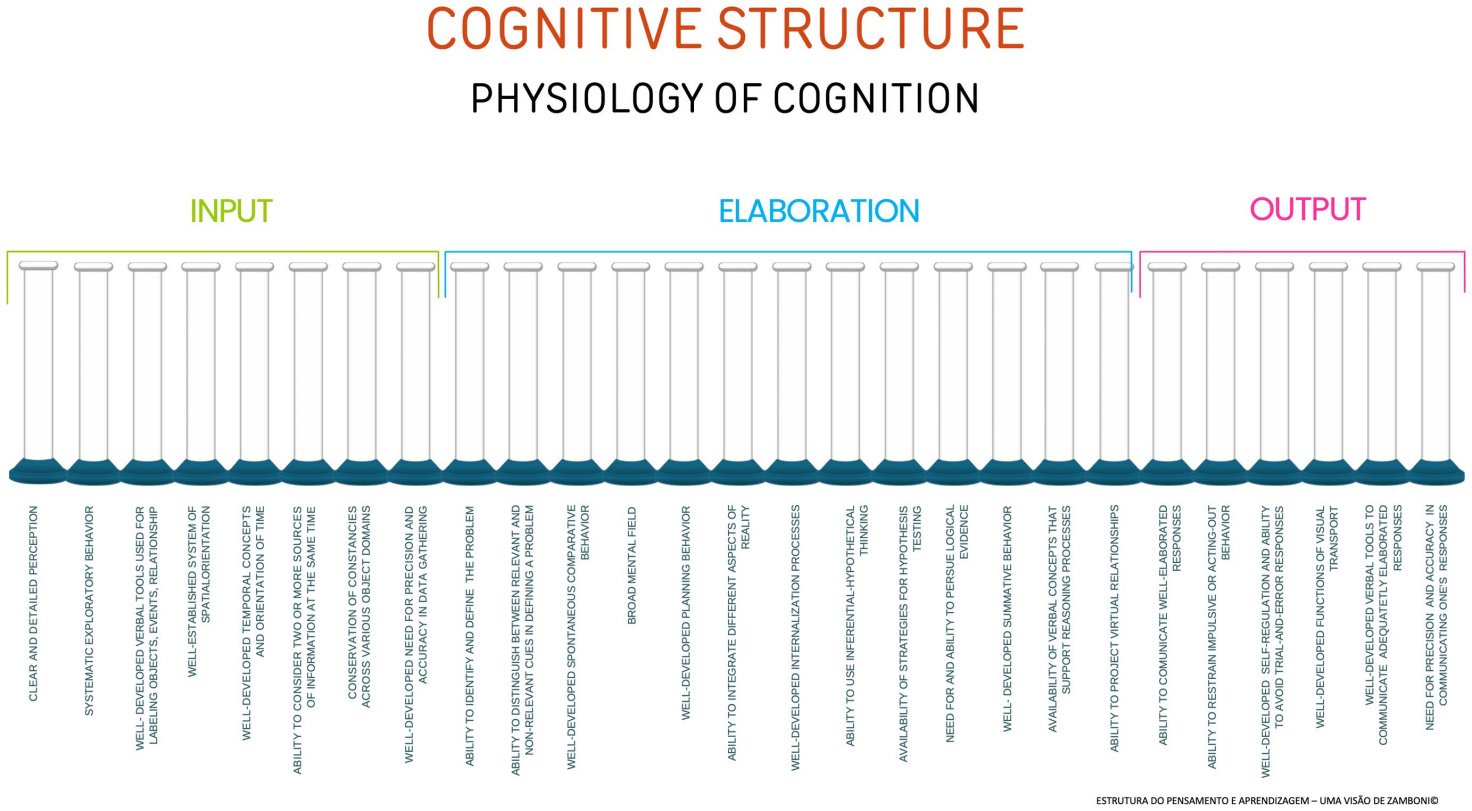
Cognitive functions depicted as tubes.

Zamboni (2024) illustrated this cognitive structure using a visual representation with cognitive functions depicted as tubes (Figure 1). These tubes are intentionally left open at the top to symbolize the limitless potential of human thought and understanding, making the concept easier to grasp.

With regard to structure, the cognitive platform is universal among individuals. However, the development, efficiency, inefficiency, or deficiency of cognitive functions vary between people, as they are influenced by various factors, such as distal etiological factors, which may be genetic, organic, or environmental stimuli, as well as proximal factors, such as the presence or absence of mediation (Feuerstein et al., 2006, p. 70).

**Figure 2:**
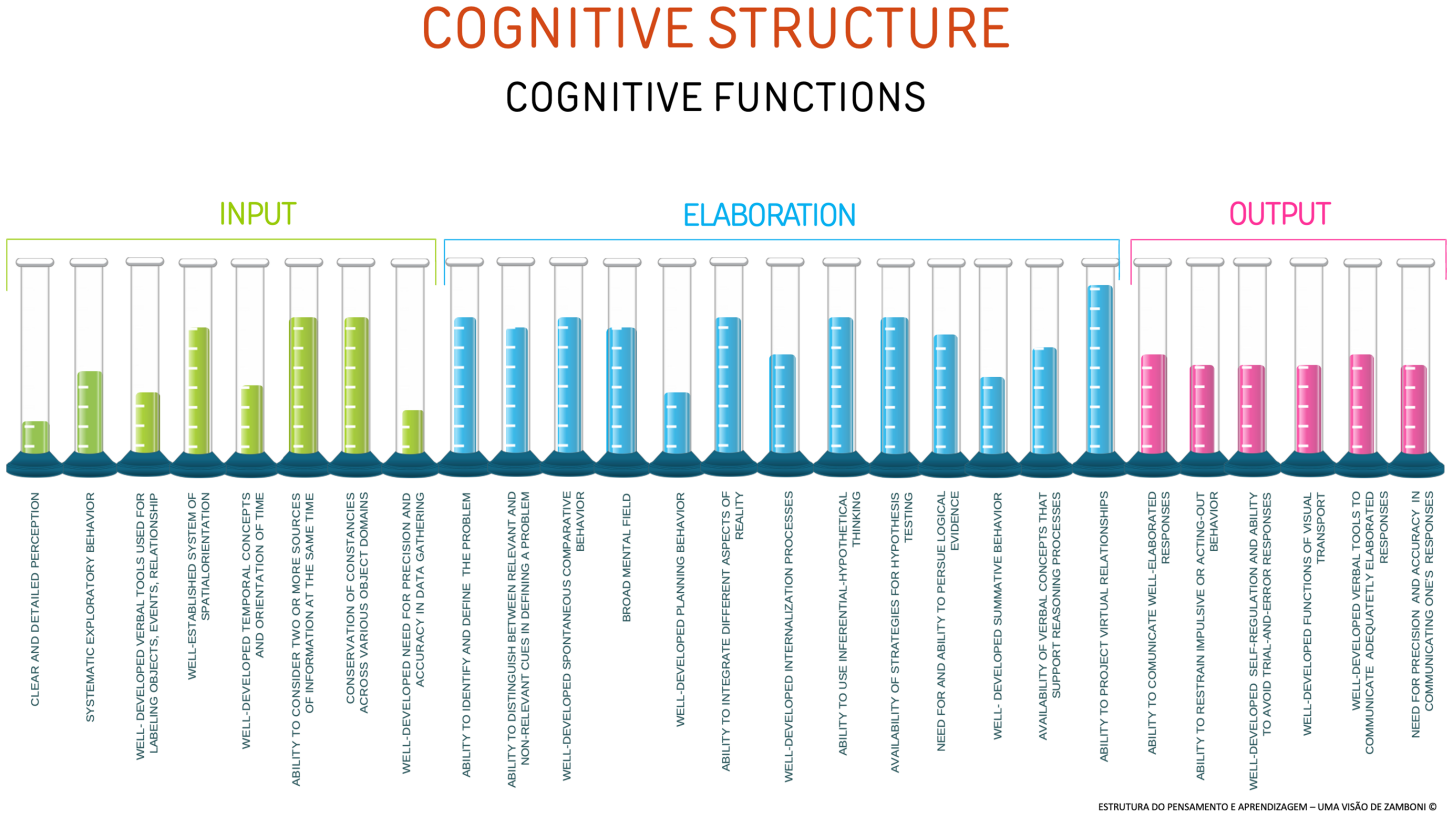
General example of varying levels of efficiency in cognitive functions.

Figure 2 illustrates a general example of a cognitive platform’s architecture.

**Figure 3:**
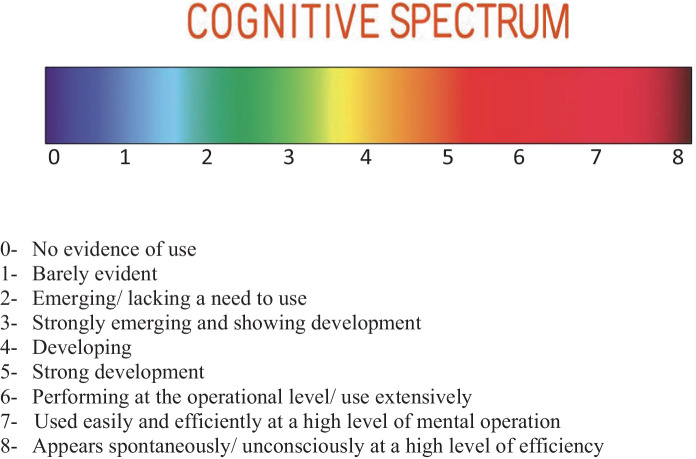
Cognitive spectrum.

The level of efficiency of the cognitive functions may be viewed as a spectrum where the cognitive functions can be efficient, register degrees of inefficiency when used ineffectively in the thought process, or even be deficient when they do not show up spontaneously in the individual’s cognitive process. The architecture of the functions may be further clarified when viewed as a cognitive spectrum (Figure 3) where each level shows a different kind of functioning corresponding to the level of efficiency (Dr. J. Zehr, personal communication, June 2024).

**Figure 4:**
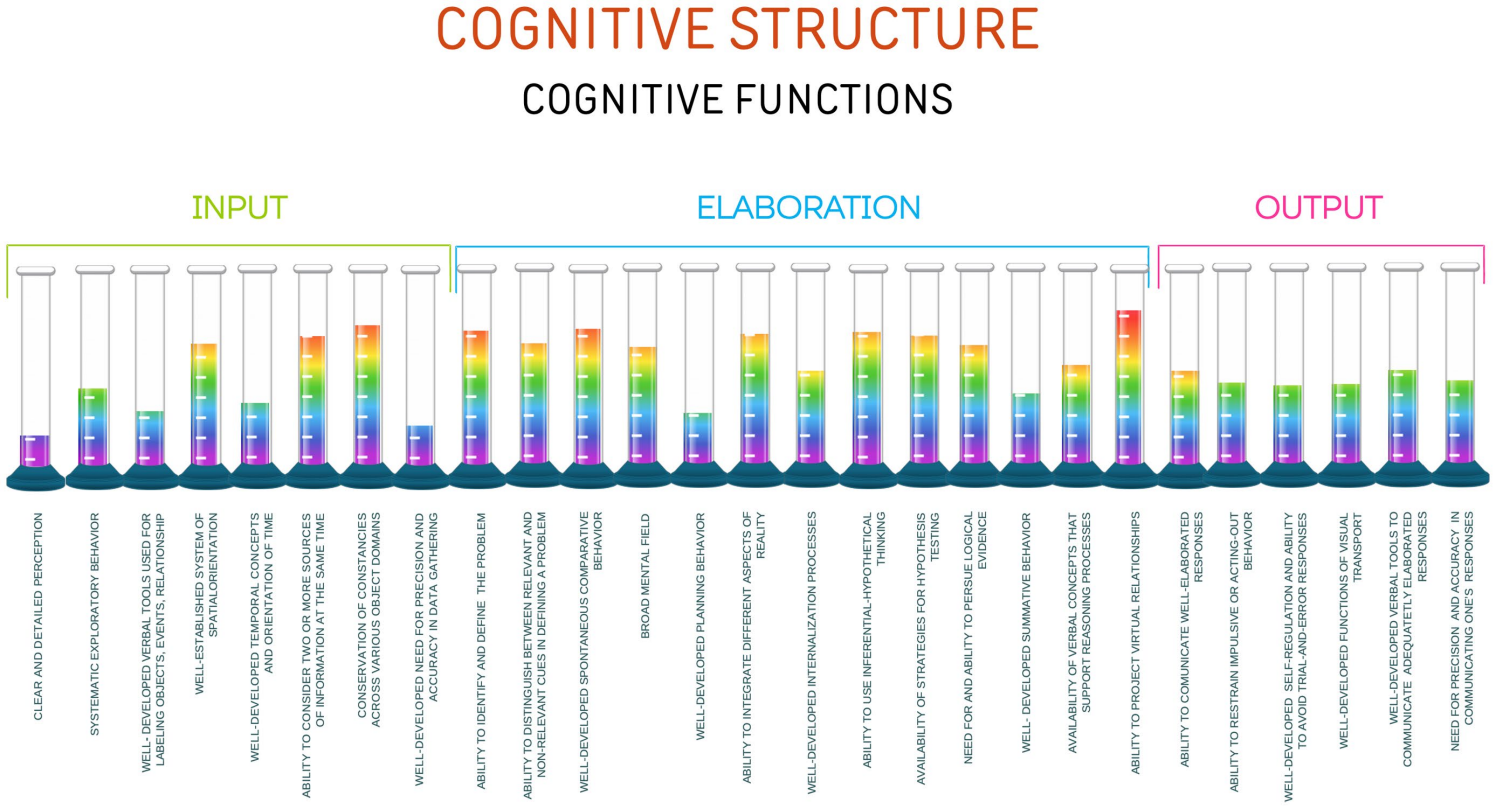
Cognitive spectrum applied to the cognitive structure.

Utilizing the cognitive spectrum, the cognitive platform may now be expanded to provide discrimination of the efficiency of the cognitive functions (Figure 4).

This cognitive framework also allows ascertaining which parts of the brain are used predominantly, as there is a correlation between the way the person thinks and the areas of the brain they prefer to access when thinking. According to Herrmann and Herrmann-Nehdi (2015): “Through increased use as a result of our preference, certain patterns of specialized thinking come to be preferred over others, developing to a higher level than the nonpreferred patterns. And as they develop over time as a result of our lives experiences, those patterns become evident from the mental preferences that we exhibit”.

From the analysis of the individual’s architecture of thinking, the processing of information can be inferred, with the potential derived from it (abilities and strengths) as well as the fragile areas that may need calibration.

## 5 Behind the thinking of attention disorder

When receiving individuals diagnosed with attention disorder, we observed that the effects were generally the same, with complaints of difficulties in focus, planning, and organization. Patterns in presentation were noticed and this prompted investigation regarding their similarities and differences. We observed that the causes, cognitively speaking, came from different origins with varying levels of efficiency among the cognitive functions. Viewing inattention disorder as a spectrum is imperative as depending on the level of efficiency, inefficiency, or deficiency of the functions and their dynamic in the structure it may differ from one individual to another. Thus, not all of those who present with attention disorder have the same level of cognitive functioning.

Presented below are the pattern of information processing observed as divided into three distinct presentations:

## 6 Architecture of thinking - Presentation A - overload of details in the thinking

### 6.1 Main characteristics

(a) The process of thinking observed was accessed in the left cortex of the brain as a first preference, according to the NBI assessment.
(b) The reports received frequently referred to the individuals as ADHD—Predominantly Inattentive Type.
(c) Processing of information:

o The input phase is normally very efficient. The information may enter the cognitive system naturally and be automatically clear and detailed, perceiving various details of the object (e.g., color, shape, size, thickness, etc.) as well as on a verbal labeling level. An efficient, systematic exploratory behavior of information in the input phase of the mental act can be observed.
o Many tend to have a strong analytical ability and sound logical reasoning, presenting a high capacity for establishing proportions efficiently among the observed data. In the elaboration phase of the mental act, the cognitive function of “the need for and ability to pursue logical evidence” is the most efficient. “Clear and detailed perception,” “systematic exploratory behavior,” “well-developed need for precision and accuracy in data gathering” are high in the input phase.
o For many, their thinking often contains a substantial amount of information that is generated automatically. As a result, they have more information to compare, contrast, and establish proportions which naturally leads to deeper and more critical thinking.
o Attention and focus may be easily lost due to the lack of establishing relevance among the information arising from an unclear or incorrect definition of the problem, which directly affects planning behavior as well as organization and focus. The cognitive function “ability to distinguish between relevant and non-relevant cues in defining a problem” in the elaboration phase of the mental act is seen as inefficient or deficient leading to a lack of coherent and sustained (over time) strategies for problem solving. When the individual cannot establish what is relevant or irrelevant, everything holds equal importance. Consequently, this inability to prioritize makes it difficult to determine a starting point. As a result, organizing tasks and creating a systematic action plan becomes an overwhelming challenge, further hindering effective time management and productivity.
o Excessive details may also compromise the efficiency of higher-order reasoning; information that is not relevant and should be ignored is often not discarded. In other words, although a conclusion may be reached, the individual continues to hold on to irrelevant information.
o It is observed that many of them complain of mental exhaustion and easy discouragement which may appear as generalized anxiety.
o This presentation showed a preference for engaging numerical (higher) and verbal modalities. The mental act can be conducted in various modalities, such as figurative, pictorial, numerical, symbolic, verbal, or combinations. The ease or difficulty of understanding something may depend on the modality in which the thinking operates on the data and the efficiency of the thinker in that modality.

**Figure 5:**
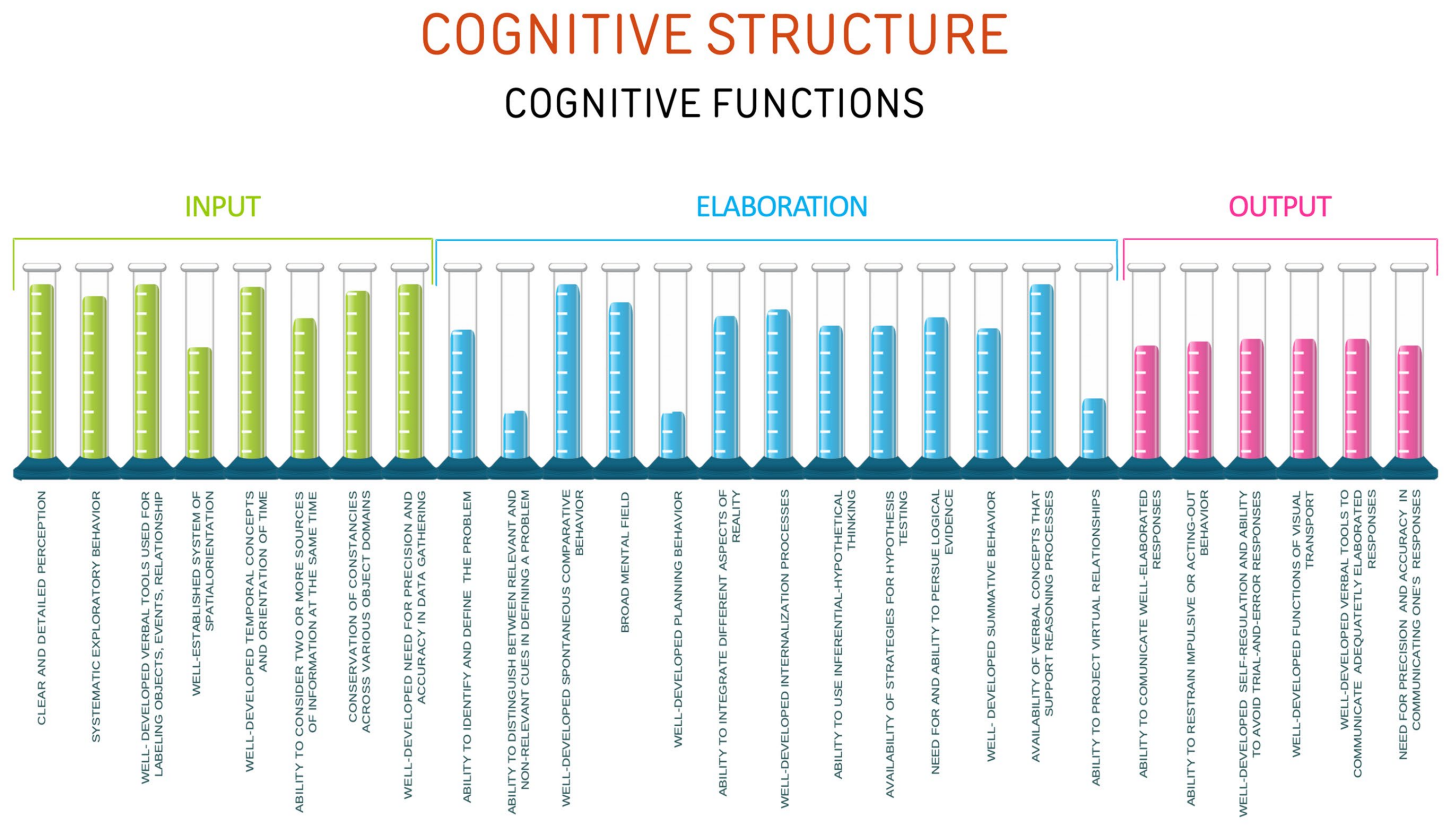
General example of Presentation A - overload of details in the thinking.

Figure 5 illustrates a general example of the cognitive platform’s functioning of Presentation A.

### 6.2 Potential

The ability to notice details allows them to access a great amount of information, promoting deep thinking. This may promote a strength of analysis.

### 6.3 Identifying the point of struggle

There are too many details in the thinking. The lack of attention occurs by getting lost among the details and not being able to classify them in order of relevance, often leading to discouragement and difficulties in planning.

### 6.4 Intervention

When thinking about a cognitive intervention, this should be planned to help the individual classify the information in order of relevance, qualified by a clear definition of the problem. This action helps to establish planning from the relevant aspects considered.

The target cognitive function is “ability to distinguish between relevant and non-relevant cues in defining a problem” in the elaboration phase of the mental act. Mediational questions can be offered to help the individual:

(1) What do I need?
(2) What is important?
(3) How do I decide what is not important?
(4) What things do I need to help do this?
(5) What is my criteria for evaluating relevance or importance?
(6) Is that a relevant part of the solution or is it irrelevant?

## 7 Architecture of thinking - Presentation B - weakened quality of details in the thinking

### 7.1 Main characteristics

(a) The process of thinking was observed to be accessed in the right cortex of the brain as a first preference, according to the NBI assessment.
(b) The reports received usually referred to the individuals as ADHD - predominantly hyperactive/impulsive.
(c) Processing of information:

o When thinking, the information that enters the cognitive system is spontaneously associated and integrated thus creating a new “whole.”
o Focus is easily lost, as they do not “stay” with the information received but instead move on to the new “whole” that has been created.
o Many possess the ability to grasp the entirety of a situation. Their thinking typically begins with an integrated perspective, moving from the whole to its individual components. During their thought process, some individuals can visualize facts in three dimensions (3D), enabling them to perceive different facets of a situation or object.
o The cognitive functions of “ability to integrate different aspects of reality” and “ability to project virtual relationships” in the elaboration phase of the mental act present a high level of efficiency, although when combined with a weakened quality of details in the input, this may result in poor accuracy in the output.
o Because of the speed in integrating data, they may frequently pass over the details, not discriminating the information clearly and precisely. In the input phase of the mental act, the cognitive functions of “clear and detailed perception,” “systematic exploratory behavior,” and “well-developed need for precision and accuracy in data gathering” were identified as inefficient.
o Many tend to present with mental flexibility. They commonly have a broader mental field, with the ability to simultaneously maintain several sources of information when thinking.
o It was observed that complex reasoning might bring difficulties due to inefficient data collection and weakened order in the sequence of the thinking process.
o This presentation showed a preference for engaging figurative and pictorial modalities. Because of this, it may be difficult to put their thinking into words (verbal modality) because this asks for the ability to decode every image/figure into words and put them in order and sequence to have a chronological order in the thinking to communicate. Difficulties in reconstructing their thinking process, either in their minds or conveying to others are observed. This is why, in some cases, they show egocentric communication (in the output phase), as they understand the idea inside their heads, however, when attempting to explain to others it may become incomplete due to synthesis as they tend to explain only the “whole” that was formed and not how they arrived there.
o Due to their high integrative association of ideas and preference for engaging figurative modality, they often struggle significantly to set a “reference point” to explain their reasoning. As a result, the new “whole” does not have a clear beginning. This results in inefficient planning behavior struggling to establish order and sequence processes.

**Figure 6:**
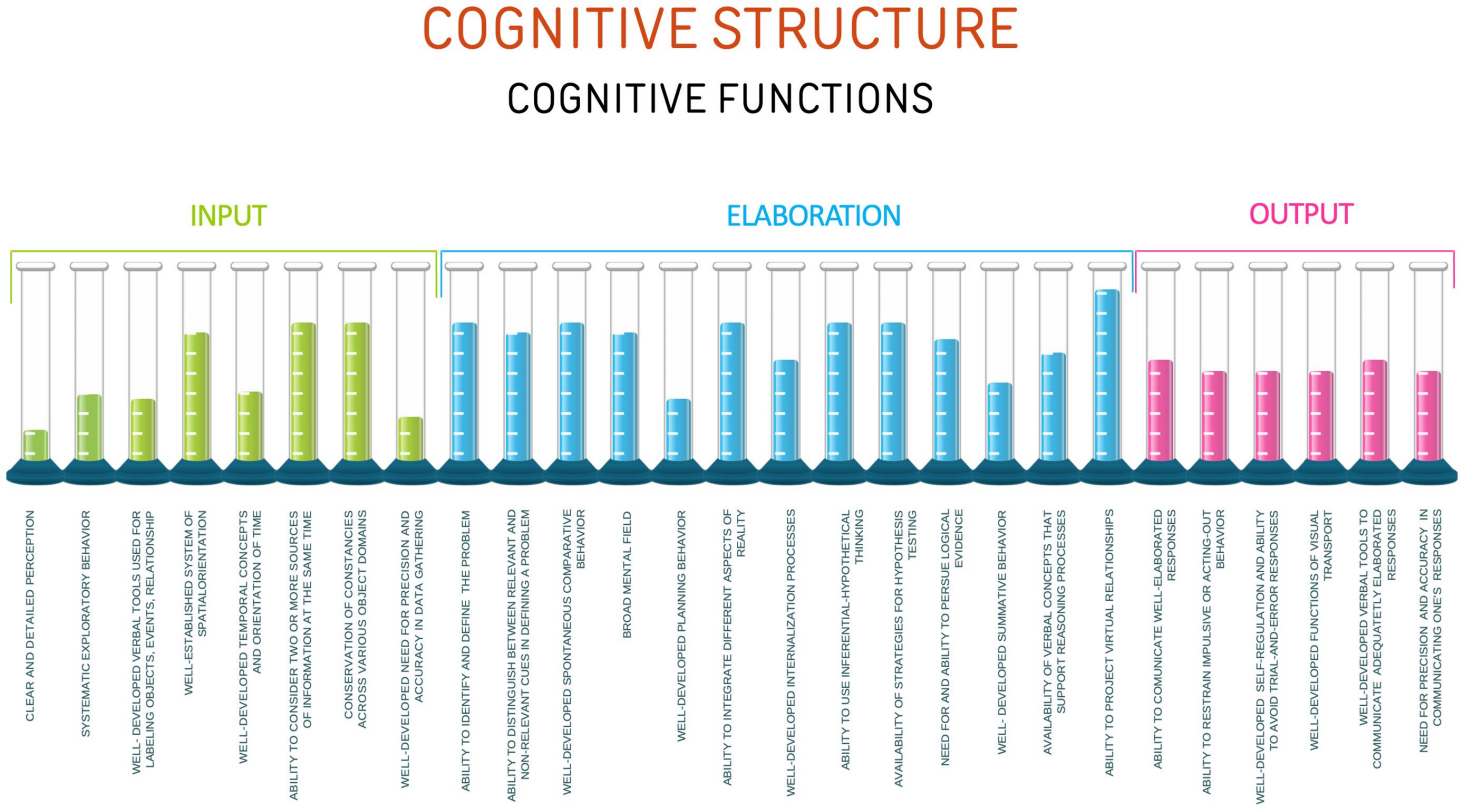
General example of Presentation B - weakened quality of details in the thinking.

Figure 6 illustrates a general example of the cognitive platform’s functioning of Presentation B.

### 7.2 Potential

Transformation, simultaneity in keeping several scenarios in mind, creativity, and movement are their high potential. The ability to find different exits or solutions to problems or situations and think out of the box is outstanding. They tend to be flexible and present with a divergent mind.

### 7.3 Identifying the point of struggle

The lack of attention occurs due to the speed of information processing, and the associative thinking that happens spontaneously. This often leads to passing over details in the collection and not discriminating the information clearly and precisely thus bringing a weakened quality of details in the thinking. Their high integrative capacity leads to efficient contextual thinking but may bring difficulties in being aware of the process that took the individual to the conclusion, affecting planning and organization.

### 7.4 Intervention

The cognitive intervention should be strategically designed to help the individual become aware of the impact of rapid thinking on their ability to gather precise and accurate information. This process involves not only fostering awareness of thinking speed but also teaching effective self-regulation techniques to moderate it during the collection of details. Clear and structured mediation is essential to enable the individual to recognize how excessive speed manifests in their behavior, such as the need for constant movement, impatience when tasks take longer than expected, a persistent sense of urgency, or the perception that engaging in certain activities is a waste of time. The intervention should challenge these tendencies through carefully designed activities, helping the individual to identify these patterns and adopt appropriate strategies for calibrating them.

Another critical aspect of the intervention is the development of linear and sequential thinking, particularly after concluding an idea. Individuals with rapid and associative thinking often lack awareness of the cognitive processes that lead them to a conclusion. While they may arrive at an endpoint, the steps taken to reach it often remain unclear, which directly impacts their ability to maintain order, establish sequence, and engage in effective planning and organization. This difficulty frequently results in uncertainty about how to begin tasks, contributing to disorganization.

To address these challenges, mediational questions can be integrated into the intervention:

1. Where should I start?
2. What is the foundation of this idea?
3. What are the underlying principles or steps that support it?
4. What comes next?
5. How do I proceed from here?

## 8 Architecture of thinking - Presentation C - struggle to keep several sources of information simultaneously in mind

### 8.1 Main characteristics

(a) The reports received usually referred to individuals as ADHD predominantly inattentive or ADHD predominantly hyperactive—impulsive presentations.
(b) Processing of information:

o The individual struggles to maintain multiple pieces of information simultaneously in their mental field of reasoning.
o The mental field is usually narrow, resulting in an episodic view of what is being thought.
o Simple reasoning is easily done. However, more complex reasoning that involves keeping several sources of information accessible becomes difficult. This happens because not all the information used to carry the reasoning is available. This affects the elaboration phase of thinking, especially the comparison process, and consequently, logical reasoning, hypothetical thinking, and the production of inferences. As a result, the individual gets lost in reasoning and may not reach a correct conclusion because of the lack of information retrievable to build efficient reasoning.
o The complaint of forgetting what has been read or heard is frequently observed.
o Parents and teachers may think the individual did not pay enough attention to what has been said or proposed; they are frequently seen as inattentive people.
o In some cases, individuals show a good level of concentration and focus when the information received is suitable to the amount they can hold in their thinking.

(c) This may also be combined with presentation type A or B, exacerbating inattention.

**Figure 7:**
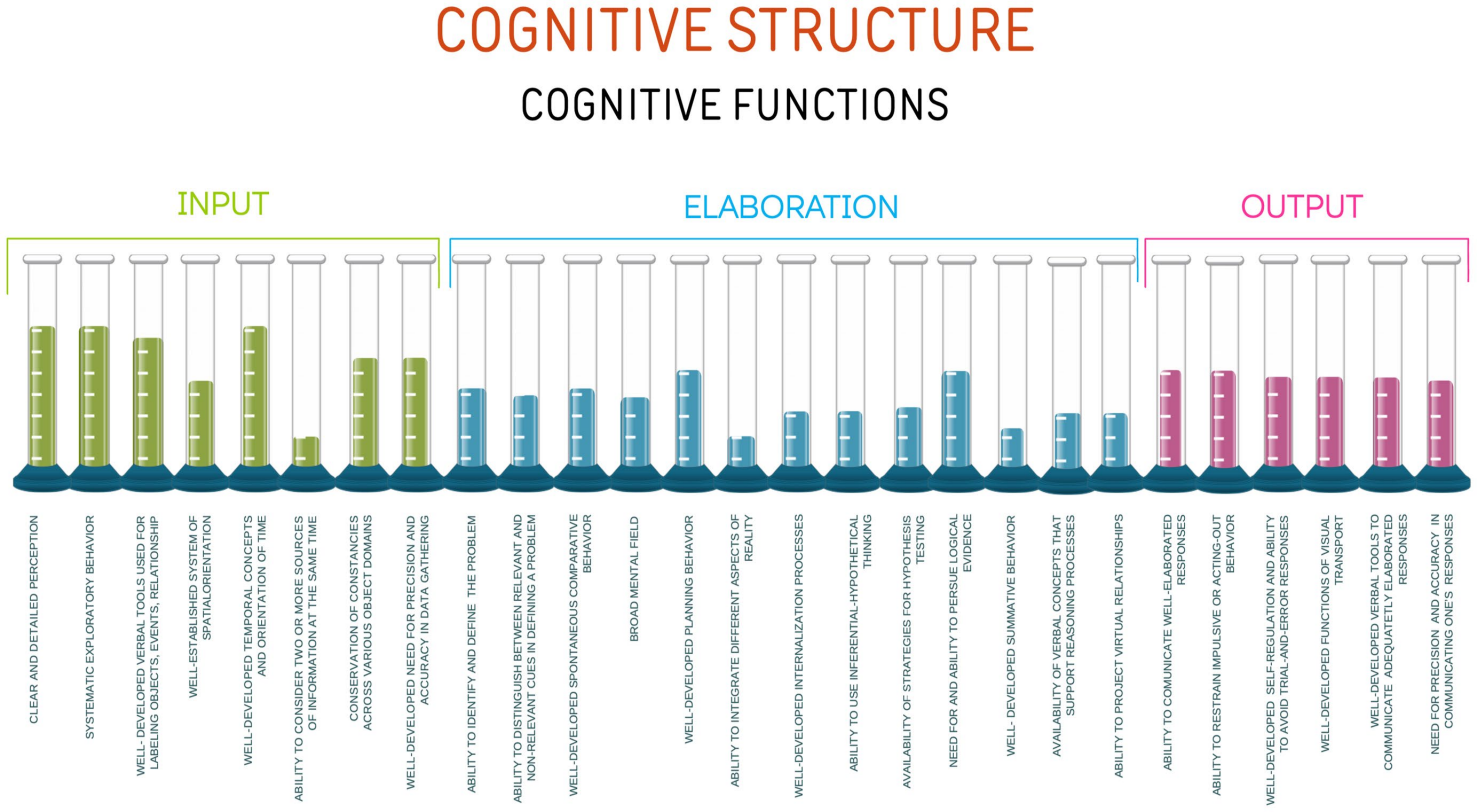
General example of Presentation C - struggle to keep several sources of information simultaneously in mind.

Figure 7 illustrates a general example of the cognitive platform’s functioning of Presentation C.

### 8.2 Identifying the point of struggle

Keeping multiple sources of information simultaneously in the mental field is difficult. The lack of attention occurs as the information does not stay complete for the purpose of comparison and integration to support reasoning, affecting the basic propensity to connect data, leading to attentional barriers (Feuerstein et al., 2006).

### 8.3 Intervention

This presentation shows a weakness focused on a specific cognitive function in the input phase of the mental act and asks for a very targeted intervention. Unlike the others, it does not demand calibration in the executive functions. According to Diamond (2013), executive functions are cognitive control processes that allow individuals to manage thoughts and actions under conditions requiring concentration and attentional control. Core functions include inhibitory control, working memory, and cognitive flexibility, which together support higher-order processes like reasoning, problem-solving, and planning.

The targeted cognitive function is the “ability to consider two or more sources of information at once.” Intervention should be planned to help the individual identify the relevant aspects of the thinking task, deciding where to pay attention, and categorizing the information. Creating sets of data and organizing principles needs to be implemented. Use of superordinate concepts and strengthening categorization skills allows the individual to hold increasing pieces of information simultaneously.

To address these challenges, mediational questions can be integrated into the intervention:

1. What superordinate concept can be used the organization of information?
2. How can I summarize what I have just read with only a few words?
3. What is the most relevant aspect that requires my attention in what I have just read?

## 9 Conclusion

Attention disorders have traditionally been studied with a focus on their neurological underpinnings and their relationship to brain function. However, this article advocates for a shift in perspective, examining attention disorders through a cognitive-behavioral and functional framework rooted in Feuerstein’s theory of Structural Cognitive Modifiability. By identifying distinct thinking patterns and their impact on both learning and daily behavior, this approach underscores the potential for tailored interventions to address the unique cognitive challenges associated with attention disorders.

A key finding of this research is the identification of a presentation of attention disorder that does not stem from inefficiencies in executive functions, as has been traditionally assumed in prior studies. This insight challenges the prevailing notion that interventions for attention deficits must always target executive functions. Instead, it broadens the scope of understanding, suggesting that in some cases, attention-related difficulties arise from other cognitive processes. This discovery is of critical importance for professionals, as it offers them an additional avenue of investigation and enables them to refine their intervention strategies to be more precise and aligned with the individual’s specific cognitive needs.

While individuals with attention disorders often exhibit difficulties in focus, planning, and organization, we recognize that these challenges stem from diverse origins. By analyzing the architecture of thinking that underpins inattention, cognitive modifiability provides a powerful tool for individuals to gain awareness of their cognitive functioning, fostering self-regulation and enhancing their capacity for change. For professionals, this understanding paves the way for more precise and effective interventions, ultimately improving outcomes for those affected by attention disorders.

This approach offers increased awareness for the individual who can then powerfully impact his effort to promote change, as understanding his cognitive functioning allows self-regulation and, consequently, self-control. This understanding is also paramount to develop a precise cognitive intervention for the professional, leading to increased efficiency of results.

Moreover, the recognition of the dynamic interplay between cognition and neural plasticity highlights the transformative potential of cognitive modifiability as a framework for mitigating the effects of inattention. This perspective not only offers practical strategies for intervention but also emphasizes the need for further research into the relationship between cognitive processes, behavior, and brain adaptation. By broadening the scope of inquiry, this approach holds promise for advancing both the understanding and treatment of attention disorders, providing a foundation for innovative and impactful therapeutic practices.

## Data Availability

The original contributions presented in the study are included in the article/supplementary material, further inquiries can be directed to the corresponding author/s.

## References

[ref1] American Psychiatric Association. (2022). *Neurodevelopmental disorders*. In: Diagnostic and statistical manual of mental disorders. Virginia, US: American Psychiatric Association.

[ref2] Centers for Disease Control and Prevention. (n.d.). *Attention deficit/hyperactivity disorder (ADHD)*. Centers for Disease Control and Prevention. Available at: https://www.cdc.gov/adhd/index.html.

[ref3] Diamond A. (2013). Executive functions. Annu. Rev. Psychol. 64, 135–168. doi: 10.1146/annurev-psych-113011-143750, PMID: 23020641 PMC4084861

[ref4] Doidge N. (2007). The brain that changes itself: Stories of personal triumph from the frontiers of brain science. New York: Viking.

[ref5] Falik L. (2019). “The relationship of cognitive modifiability to neural plasticity: from the Feuerstein perspective” in Advances in mediated learning experience for 21st century education: Competencies, contexts and culture. eds. Tan O. S. Bee L. C. Wong Y. F. I. (Cengage Learning Asia: Singapore).

[ref6] Feuerstein R. (1980). Instrumental enrichment: An intervention program for cognitive modifiability. Baltimore: University Park.

[ref8] Feuerstein R. Feuerstein R. S. Falik L. H. (2015a). LPAD examiner’s manual: The dynamic assessment of cognitive modifiability learning propensity device standard. Israel: The Feuerstein Institute.

[ref9] Feuerstein R. Feuerstein R. S. Falik L. H. (2015b). Changing minds and brains: The legacy of Reuven Feuerstein: Higher thinking and cognition through mediated learning. New York: Teachers College Press.

[ref10] Feuerstein R. Feuerstein R. S. Falik L. H. Rand Y. (2002). The dynamic assessment of cognitive modifiability: The learning propensity assessment device: Theory, instruments and techniques. Jerusalem: ICELP.

[ref11] Feuerstein R. Feuerstein R. S. Falik L. H. Rand Y. (2006). The Feuerstein instrumental enrichment program: Revised and expanded edition. Jerusalem: ICELP.

[ref7001] Herrmann N. (1988). The creative brain. Brain Books.

[ref12] Herrmann N. Herrmann-Nehdi A. (2015). The whole brain business book: Unlocking the power of whole brain thinking in organizations and individuals. 2nd Edn. New York: McGraw-Hill Education.

[ref13] Kleim J. A. Jones T. A. (2008). Principles of experience-dependent neural plasticity: implications for rehabilitation after brain damage. J. Speech Lang. Hear. Res. 51, S225–S239. doi: 10.1044/1092-4388(2008/018), PMID: 18230848

[ref14] Monastra V. (2022). *What is neurofeedback?* A Brain-Training ADHD Treatment. ADDitude. Available at: https://www.additudemag.com/basics-neurofeedback-for-adhd/.

[ref15] National Institutes of Health. (2024). *NIH researchers identify brain connections associated with ADHD in youth*. Available at: https://www.nih.gov/news-events/news-releases/nih-researchers-identify-brain-connections-associated-adhd-youth

[ref16] Nigg J. (2024). *Beyond genes: leveraging sleep, exercise, and nutrition to improve ADHD*. Additude. Available at: https://www.additudemag.com/adhd-lifestyle-changes-food-sleep-exercise-genes-environment/

[ref17] Piaget J. (1952). The origins of intelligence in children. New York: International Universities Press.

[ref18] Psychology Today. (n.d.) *Neuroplasticity*. Available at: https://www.psychologytoday.com/us/basics/neuroplasticity.

[ref20] Zamboni D. (2024). *Estrutura do pensamento e aprendizagem: uma visão de Zamboni*. Registro intelectual 899.408, Biblioteca Nacional.

[ref9001] Zehr J. Bergeron L. (2019). Another way for ADHD: Application of The Feuerstein Program as a Non-Pharmacological Approach to Improving Symptoms of ADHD.

